# Association of Drugs for Sale on the Internet and Official Health Indicators: Darknet Parsing and Correlational Study

**DOI:** 10.2196/56006

**Published:** 2024-11-15

**Authors:** Sergey Soshnikov, Svetlana Bekker, Bulat Idrisov, Vasiliy Vlassov

**Affiliations:** 1 Public Health Division School of Health Scienses Central Michigan University Mount Pleasant, MI United States; 2 Higher School of Economics Centre for Health Economics, Management, and Policy National Research University Moscow Russian Federation; 3 Department of Health Systems and Population Health School of Public Health University of Washington Seattle, WA United States; 4 National Research University Higher School of Economics Moscow Russian Federation

**Keywords:** darknet, Internet black market, illicit drugs, Hydra, marketplace, cannabis, opiates, zakladka, Bitcoin, crypto, public health, overdose, harmful drug use, drug availability, drug use

## Abstract

**Background:**

Studying illicit drug circulation and its effects on population health is complicated due to the criminalization of trade and consumption. Illicit drug markets have evolved with IT, moving digital to the “darknet.” Previous research has analyzed darknet market listings and customer reviews. Research tools include public health surveys and medical reports but lack neutral data on drugs' spread and impact. This study fills this gap with an analysis of the volume of drugs traded on the darknet market.

**Objective:**

We aimed to use the dark web data and officially published indicators to identify the most vulnerable regions of Russia and the correlations between the pairs of variables to measure how illicit drug trade can affect population well-being.

**Methods:**

We web-parsed the Hydra darknet drug marketplace using Python code. The dataset encompassed 3045 individual sellers marketing 6721 unique products via 58,563 distinct postings, each representing specific quantities sold in different Russian regions during 2019. In the second stage, we collected 31 variables from official sources to compare officially collected data with darknet data about amounts and types of selling drugs in every 85 regions of Russia. The health-related data were obtained from official published sources—statistical yearbooks. Maps, diagrams, correlation matrixes, and applied observational statistical methods were used.

**Results:**

In 2019, a minimum of 124 kilograms of drugs circulated daily in small batches on the Russian darknet. Cannabis dominated the market, being 10 times more prevalent than opiates, and cannabis products' higher availability in the region is correlated with a lower incidence of opiate overdoses. The “grams of opiates in the region” variable is significantly correlated with drug overdose deaths (*r*=.41; *P*=.003), HIV-positive cases due to drug use (*r*=.51; *P*=.002), and drug court convictions in Russia (*r*=.39; *P*=.004). The study identified significant correlations between opiate sales on the darknet and higher rates of HIV among injection drug users (*r*=.47; *P*=.003). Conversely, regions with higher cannabis sales exhibited significant negative correlations with indicators of harmful drug use (*r*=–.52; *P*=.002) and its prevalence (*r*=–.49; *P*=.001). These findings suggest regional variations in drug sales on the darknet may be associated with differing public health outcomes. These indicators accurately reflect regional drug issues, though some official statistics may be incomplete or biased.

**Conclusions:**

Our findings point to varying levels of risk associated with different types of drugs sold on the darknet, but further research is needed to explore these relationships in greater depth. The study's findings highlight the importance of considering regional variations in darknet drug sales when developing public health strategies. The significant correlations between drug sales data and public health indicators suggest that region-specific interventions could be more effective in addressing the diverse challenges posed by illicit drug use.

## Introduction

### Background

The volume of illicit drug circulation on the narco-scene depends on the market availability, local culture, specific drug effects, and legal status of the substance. It makes the research on substance use intrinsically problematic. Surveys, death certificate data, and medical reports are the principal sources for studying substance use problems. Unfortunately, there is neither neutral nor complete information in medical sources on how this drug is spread and what harm it brings to the communities. Therefore, thorough triangulation of the estimates supplied by studies of multiple designs is needed to produce accurate estimations. For example, the study of illicit drug use in juvenile justice [1] explores the high co-occurrence of conduct disorder symptoms with illicit drug use in youth involved in crime.

Illicit drug markets use communication technology, cryptocurrency, blockchain, and money laundering to avoid legal repercussions [2]. Nowadays, they have relocated online, especially to the more clandestine areas of the “darknet.” In 2022, there were 133 publications returned when the phrase “darknet AND drugs” was used in the search in MEDLINE. These publications are primarily based on data gathered through passive observation. Some researchers offer cryptocurrency transaction monitoring, with a tiny portion offering data documented from the initial request for a drug purchase through the final qualitative examination of all received samples from 3 vendors on the “dream market” darknet marketplace [3]. Some authors attempted to analyze a single substance using “Big data” techniques [4]. Recent projects have scanned a variety of digital markets to gain a better knowledge of these markets' offers, country disparities, and availability of specific substances. Interviews with merchants and consumers are a crucial part of understanding the mechanisms of the Internet market [5-8].

The role of the darknet in the drug trade has substantially grown over the last decade. With the advent of the Silk Road, the first darknet marketplace, customers and vendors have collaborated to disseminate and acquire goods and services that were previously unavailable digitally [5,6]. Numerous studies have explored the growth of these anonymous marketplaces and their role in the cybercrime ecosystem [9-15].

In a comprehensive study, the European Monitoring Centre for Drugs and Drug Addiction and Europol [9,16] discussed various perspectives for enforcement, research, and policy regarding drugs and the darknet. On the academic front, studies have focused on analyzing the content of anonymous market listings and forum posts associated with the darknet opiate trade; its pathways, risks, and rewards associated with selling drugs on darknet markets were the subjects of these studies [10,17,18]. Some researchers focused on anonymous digital marketplaces' customer reviews [12,19]. Jardine Lindner and others [20,21] conducted a study design using a big data research design to gain insights into the dark web's impact on cannabis use in the United States.

The region-specific data on the darknet drug trade, particularly in Russia, has been sparse. Our research aims to fill that gap by presenting a comprehensive, quantitative analysis of the average number of diverse types of drugs traded on the darknet per 100,000 people across different Russian regions. We explored the intricate nexus between the estimated magnitude of the illicit drug market, derived from data from a significant Russian darknet market, Hydra (at least 69% coverage of the Russian population) [22]. The traditional and new ways of distribution social networks and messengers like Telegram and WhatsApp are not covered in this study.

### The Analysis Steps

The steps undertaken in the analysis are outlined in Textbox 1.

**Analysis steps.**

- Quantifying the overall amount and types of drugs offered by the anonymous digital marketplace, thus gaining an in-depth understanding of the scale and variety of the drug trade.
- We evaluated the relative frequency of drug offers, providing insights into the persistence and repetitiveness of these illicit activities, which can indicate market demand and supply dynamics.
- Conduct region-wise research on illicit drug market activities, discerning patterns, and discrepancies in regional drug prevalence. This enables us to draw a detailed map of drug trade hotspots across Russia, considering both the urban and rural landscape.
- The metadata collected on drug-related consequences indicators from official legal, police, and health-related data sources is helpful in our approach. It can be useful for combining darknet data for further analysis and finding matches between health-related indicators and officially recognized indicators of illicit drug use disorders.

This is why the study's primary purpose, like Yannikos and other studies [23-25], was to define it as bridging public health and the illicit drug market. By analyzing the scale and nature of drug availability, the frequency of offers, and the regional distribution of illegal drug activities on a Hydra market, we aim to link these findings with official indicators of illicit drug use disorders. We integrated metadata from official legal, police, courts, and health-related variables with darknet data for a comprehensive analysis but did not use apps as a data source as in another research [26]. Having outlined the previous investigations and the significance of developing the new darknet drug markets research methodology, we now turn to the methods employed to investigate these issues.

## Methods

### Data Collection

#### Darknet Parsing

The list of the offered on-the-market drug data was gathered from the “darknet market” Hydra, along with the volume delivered and region of the offer. The data were collected using advanced web scraping techniques to extract specific information on drug types, quantities, and corresponding regions. These platforms, operating within the Onion Router (TOR) network (a network that allows anonymous browsing), are anonymous and transient; therefore, specific market names are omitted.

**Figure 1:**
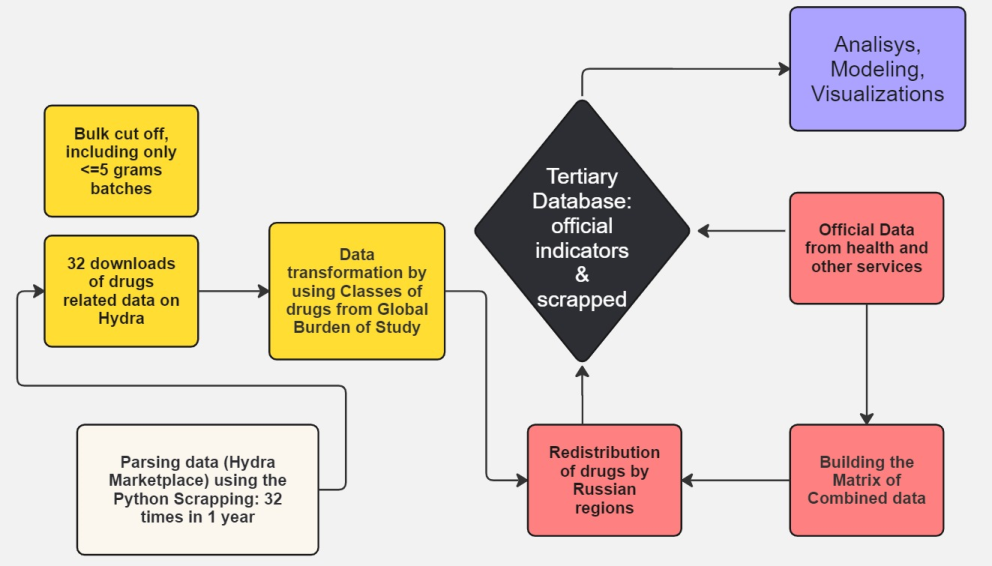
Data collection, combination, and analysis flowchart.

In the next step, we have meticulously gathered official data on Russian regions from the Ministry of Health of Russia, police reports, court reports, and the prison system [27]. Figure 1 presents the comprehensive cycle of collecting, modifying, and analyzing data at every step, ensuring the utmost accuracy and reliability. The pathway of the data collection, cleaning, transforming, classifying by drug type, and disaggregating by the regions is presented in Figure 1.

The data collection and analysis steps are presented on the flowchart in Figure 1 and explained below. The web-scraping of Hydra was conducted over a year 32 times. These 32 complete Hydra marketplace downloads were performed 2-3 times monthly. Thus, we could estimate the dynamics of market scene changes during a relatively extended period. This way of selling drugs can be explained by the fact that most of the supplies of drugs on Hydra are provided in a non-contact way—by using unique hidden places with photos of places of remote drugs and geospatial coordinates that are later offered to the buyers of drugs using the TOR and Onion browser as well. Users and sellers use the cryptocurrency “Bitcoin” to complete their deals. All these conditions theoretically support the anonymity and safety of the business. The mandatory barring of leaving houses for people during the COVID-19 disaster limited the supply of drugs in Russia. We do not have monthly data on drug poisoning, but we found significant correlations with this variable in yearly data.

While Hydra specified a category for each listing, we decided to further categorize all listings into 5 categories, aligning with the categorization offered by the Global Burden of Disease study. This adaptable approach ensures that our results can be harmonized for future comparison, providing a comprehensive understanding of the drug market dynamics.

We carefully considered the data structure and conducted spatial analysis to ensure a robust analysis. In our research, we included only the listings containing less than or equal to 5 g of any substance, thereby eliminating outliers and wholesale listings. Listings measured in units or milliliters were also excluded due to the difficulty distinguishing personal purchases from wholesale ones. In total, 3045 individual merchants were selling 6721 unique items and 58,563 unique listings where specific amounts of products were sold in a particular region of Russia. At the same time, the study's findings represent a specific time frame and may not capture evolving trends in drug use and market dynamics.

#### Official Statistics Data

Health-related data from officially published sources. We conducted an extensive analysis of 31 official sources to assess the relevance of various indicators to drug sales on the darknet market Hydra in all 85 regions of Russia. Our data was sourced from a range of authoritative statistical reports, including “Socially Significant Diseases of the Population of Russia in 2019” by the Federal Research Institute for Health Organization and Informatics of the Ministry of Health, “Activities of the Narcological Service of the Russian Federation in 2017-2018 (Analytical overview)” by the V. Serbsky National Medical Research Centre for Psychiatry and Narcology, “Report on Drug Situation in the Russian Federation in 2018” by the Russian State Anti-Drug Committee, and “51-C” Form by the Russian Federal State Statistics Service—Rosstat. The references to the primary data sources are available in the table “Russian Drug Use Disorders Database” [27]. All the variables that were collected for this research are presented in Table 1.

**Table 1:** The variables used in the correlation matrix in the study were related to drug use-related problems in 2019 (all variables are relative).

	Name	Description
1	popul	The population of regions of Russia
2	incdrugdep	Drug dependence incidence
3	incharmuse	Harmful drug-use prevalence
4	prev inject	Injecting drug users prevalence
5	HIVIDU	Number of HIV-positive IDUs^a^
6	hivamongidu	Percent of HIV positive among registered IDUs
7	incinjdrug	Incidence of injection drug use
8	incinjpercensocialsign	Relative rate of patients who inject drugs (rate among incidence)
9	prevalsubs	Prevalence of people with substance use (drugs and alcohol)
10	drugpreldrug	Drugs dependence prevalence (no alcohol)
11	drugprrelharm	Harmful drug-use prevalence
12	inchiv	HIV incidence overall (not specific to drug users)
13	prevhiv	HIV prevalence overall
14	Instit	Number of multidisciplinary institutions with outpatient narcological departments (offices)
15	beddays	Bed occupancy (days per year)
16	res5	The average length of stay of the patient in the bed
17	res6	Duration of treatment (days per patient)
18	narcotest1	The number of schoolchildren and students tested for drugs
19	reftest	The number of schoolchildren and students who refused to test
20	reftestrel	The number of schoolchildren and students who refused to test
21	postest	The number of schoolchildren and students testing positive for drugs
22	postestrel	The number of schoolchildren and students testing positive for drugs
23	drugtraf	The involvement of drug users in illicit trafficking 2018
24	narcourt	The criminality of drug addiction (the effect of drug addiction on drugs situation) 2018
25	dr_conv	The proportion of humans of drug-related crimes to all humans with crimes
26	y_drconv	The proportion of youth (below 18 years of age) convicted for drug use among all convicted for drug use
27	socrprev	Prevalence of drug use based on social studies (percent in a cohort)
28	prevpolice	Prevalence of drug users based on police data
29	deathp	Death from drug poisoning based on pathology report
30	opioidprev	Opiate dependence prevalence (registered)
31	cannabprev	Cannabis dependence prevalence (registered)
32	opioidinc	Opiate dependence incidence (registered)
33	cannabis	Cannabis dependence incidence (registered)

^a^IDUs: injection drug users.

The data and more detailed information about every variable are available through the references [27,28]. Not all official variables were matched with parsed variables, so we rejected them for further analysis but kept them in Table 1 to demonstrate how many hypothetical matches were tested.

### Data Analysis

#### Overview

In the last stage, we analyzed comparisons of its distribution across areas to assess the public health burden of drug impact. For this step, the statistical methods include observational statistics, building diagrams, and mapping by the regions of Russia. We implemented Spearman correlations analysis to find and measure levels of associations between darknet marketplace and official health statistics variables. We recognize that there may be a temporal lag between the sale and distribution of drugs under certain conditions. Nonetheless, we also considered the inertial nature of the trends and processes in the pharma market when deciding to implement this strategy with this minor design flaw.

#### Data Parsing and Categorizing

During our analysis, we undertook the following data manipulation steps for 2019, although data were available for both 2019 and 2020. Using the Python parser, we aggregated 21 data extracts from 2019 for the whole of Russia. Then, the dataset was refined to align with the research goals. We filtered out the data entries to include only those quantities specified in grams rather than pieces or milliliters. After that, we retained those designated “for personal use,” which we defined as equal to or less than 5 g. Each data entry was assigned one of 5 drug categories: opiates, cannabis, amphetamines, cocaine, or others. Within each data extract, we calculated the total sum of grams for all drugs and each category of interest (opiates, cannabis, and all drugs excluding cannabis) per region. These sums were then adjusted by dividing them by the population size of the respective regions. The data were compiled into a table consisting of 85 rows representing the regions and 22 columns representing the adjusted sums of drugs in grams per region at a single time point. Finally, a mean value was computed for the variable “grams per region at a single time point” across all time points—21 in total—providing us with an average for our analysis.

The data collection and analysis were subject to various analytical procedures used and implemented to provide a detailed understanding of the prevalence of the hidden drug “zakladka” and its impact on population health across Russia and every region. Descriptive statistics were implemented, and the initial analysis involved calculating the average amounts of different drugs per 100,000 population across all regions.

We have built the interactive maps and conducted the preliminary visualizations to construct the hypothesis. This provided a baseline understanding of the spread and prevalence of various drugs.

In the next step, a comparative analysis across different Russian regions was performed to understand the variations in drug trade patterns. This nonparametric approach was chosen because it can distinguish between linear and non-linear connections, giving the data a more nuanced perspective.

Data visualization and imagining approaches were used to help interpret the results of this study step. Diagrams and pictures were created using Python’s Matplotlib and Seaborn libraries [29] to illustrate the patterns and trends across different regions and for other drug types.

**Figure 2:**
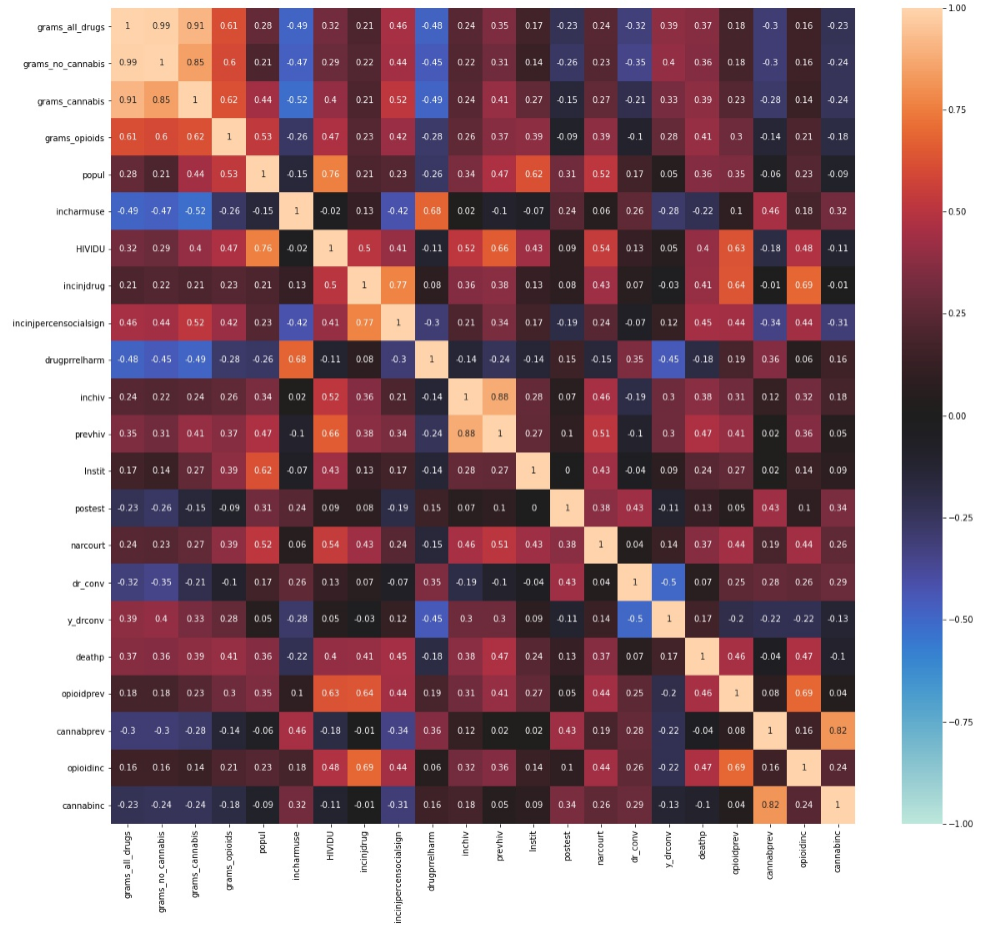
Correlation matrix of Hydra marketplace indicators with official statistical variables.

The Spearman correlation was implemented for all lists of variables, and all significant and relevant correlations were assembled into the matrix of correlation coefficients. The pairs of significant correlations are reflected in Figure 2. The only significant correlations were left for the correlation matrix and presented in this figure.

### Ethical Considerations

The research did not require the use of human participants or private information that could identify individuals. All data analyzed were aggregated, public data, hence protected against the collection or processing of personal data or any identifiable information. The research was conducted while keeping a strict standard on data privacy to protect individual privacy throughout this study.

Data for the study are publicly available from the Hydra darknet marketplace and from official Russian health statistics. The use of these datasets is in accordance with ethical standards for research involving publicly available information.

The research used appropriate sources of data and effective practices to analyze the data to secure its validity and reliability. This study was conducted using Central Michigan University's policies and procedures. The study did not require ethics approval because it did not involve human subjects. Nevertheless, the research was performed according to institutional requirements for ethical research practice.

This paper recognizes that the data it uses originates from the darknet, a virtual illicit market. The research team took all protective measures to avoid any active participation in illegal activities, but the data analysis was strictly for academic purposes, which would enrich scientific knowledge in the field of public health. It did not include participating in or advocating an illicit activity.

The authors have no conflicts of interest to declare. The research has been conducted solely for academic and public health purposes. No commercial or personal interests of the authors can steer the study or its outcomes.

## Results

As we were focused on understanding the market structure better, we tested several hypotheses and gained the following results (Textbox 2).

Table 2 represents the 15 regions with the highest volumes of all drugs in Russia. A full table with all regions is available in Multimedia Appendix 1. Central and Eastern regions are most affected; the leaders are Moscow and Saint Petersburg cities and oblasts.

**Figure 3:**
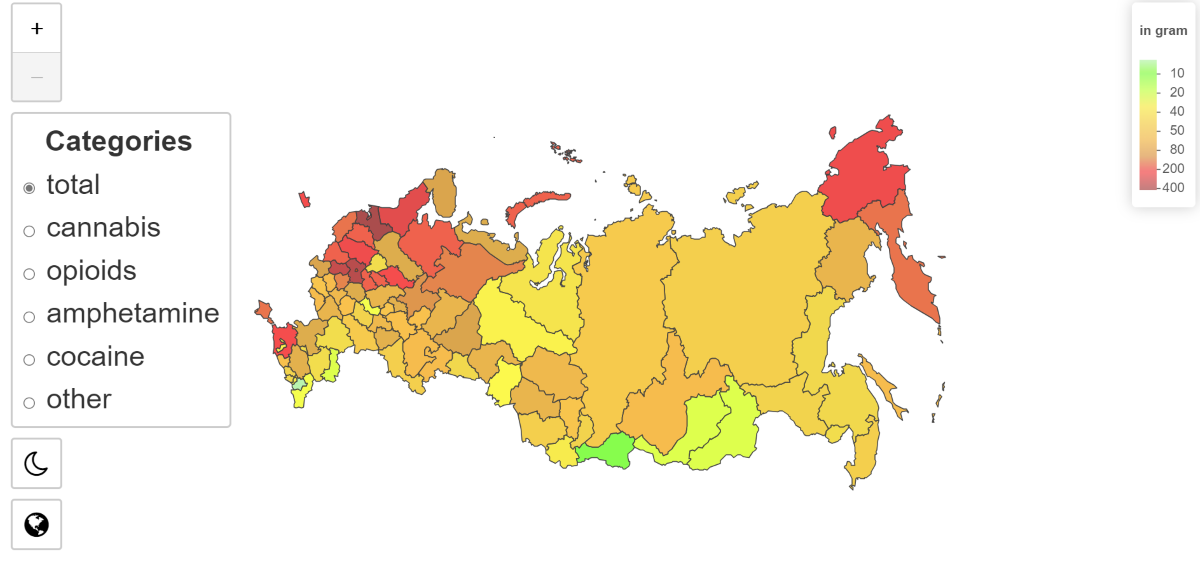
Regional distribution of all drugs for sale presented on Hydra, in grams per 100,000 population in Russia.

The map in Figure 3 illustrates the daily supply volumes of total amounts of drugs, measured throughout the year and brought to average. The link shows more detailed visualizations and data on our GitHub [29].

The map indicates the grams of drugs (cannabis, opiates, cocaine, stimulants, and other drugs) per 100,000 population. This means all these supplies are ready to buy and available in hidden secret places named “zakladka.”

We took only “zakladka” with a small weight of substances ready for sale (5 g) because larger supplies can be resold on the same Hydra marketplace by other drug dealers so that they could be counted twice.

In absolute numbers, the total amount of drugs in supplies equal to or less than 5 g was prepared for sale in amounts equal to 123,486 g (about 272.24 lb). This is the weight of drugs ready to buy in Russia on an average day in 2019. The leading regions: Moscow suburb region has 20,022 g (about 44.14 lb) observed at one moment; Moscow city, 18,998 g; Saint Petersburg, 7190 g; Krasnodar, 7162 g; Leningrad oblast, 5325 g (about 11.74 lb) of hidden drugs for sale daily.

The following graphs show the average amount of grams of psychoactive substances in a region adjusted per 100,000 population. The averages were obtained by averaging the data across all samples to get a more accurate result and eliminate the possible influence of seasonality, weekends, holidays, etc.

**Figure 4:**
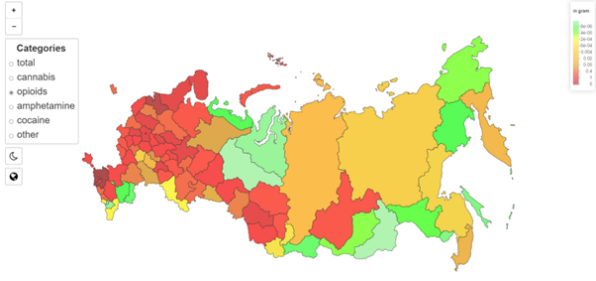
Regional distribution of opioids for sale presented on Hydra, in grams, per 100,000 population in Russia.

Figure 4 illustrates the distribution of opiates by region, setting the stage for the subsequent discussion on regional public health impacts.

The map in Figure 4 demonstrates the daily supply volumes of opiates, measured throughout the year and brought to average. The map indicates the grams of drugs (opiates) per 100,000 population. Looking at this picture, we can say that opiate supply is most common in the Northern Caucasus and Southern Siberia regions and Central Russia and less common in the “rich” regions. Large cities such as Moscow and Saint Petersburg are also affected more by opiates, as they are the gates for the drug flow into the country. The less populated regions of Siberia are less affected by opiates in Russia.

Figure 2 presents the correlation matrix of Hydra marketplace indicators with official statistical variables. This matrix is a crucial tool in our research, as it helps us identify and understand the relationships between these variables.

Each cell in the matrix shows the correlation between 2 variables, and the color scale reflects the strength and direction of the correlation, with warmer colors like red typically indicating a stronger positive correlation and colder colors like blue indicating a stronger negative correlation. The most significant correlations were observed for the variable “grams opiates” because the official statistics primarily include opiate use and overdose. As a result, the highest correlation between this variable, which can be interpreted as “grams of opiates in the region,” is strongly associated with the rates of deaths from drug overdose (*r*=.41; *P*=.003) and the number of HIV-positive people infected by drug use (*r*=.51; *P*=.002). HIV due to injected drug use has a strong correlation with amounts of opiates for sale (*r*=.47; *P*=.003). More opiates for sale mean more drug court convictions in Russia (*r*=.39; *P*=.004). At the same time, if more cannabis is for sale, then there is less incidence of harmful drug use (*r*=–.52; *P*=.002) and less prevalence of harmful drug use (*r*=–.49; *P*=.001). This means that these official indicators reflect the actual situation in regions very well, so it is worth noting that some official statistics might be incomplete or biased. A significant correlation coefficient was measured between the variables “grams of opiates” in the region and the “number of convicts for possession and distribution of drugs” (*r*=0.42; *P*=.002). More correlations can be found in Figure 2.

**Results for tested hypotheses.**

- Over half of vendors sell their products within one region. Fewer vendors sell their products in more than 5 regions. Thus, most shops tend to cover a smaller area to distribute drugs.
- More than half of all vendors sell drugs from one category, and some of them sell products from 5 categories. This means that stores are trying to focus on one type of drug rather than being one-stop sellers of all kinds of drugs.
- Of the 4 main drug categories of interest, cannabinoids are the most popular drug for sale on the dark web. The following categories in terms of popularity are amphetamines and cocaine, while opiates account for only a tiny part of the dark market. This is similar to Soska and Christin's and others' research results [6,11,12,30,31]—the most common drug is cannabis, while the least popular category is opiates.
  We used data from the Hydra dark marketplace for the following substances and their combinations to build the maps (Figures 3 and 4) during the research challenging these variables (Textbox 3).

**Table 2:** The average grams of drugs in the 15 ranged regions presented by the Russian region per 100,000 population.

	Region	All drugs	Cannabis and its products	Drugs without cannabis	Opiates
1	Leningrad region	401.68	88.27	313.41	19.51
2	Moscow region	342.60	88.70	253.90	8.74
3	Kaluga region	287.93	70.40	217.53	8.48
4	Saint Petersburg	276.83	71.87	204.96	10.26
5	Republic of Karelia	250.11	62.97	187.14	10.97
6	Tver region	222.24	52.22	170.02	7.60
7	Chukotka	221.68	52.08	172.42	18.49
8	Kaliningrad region	209.60	71.24	138.36	6.04
9	Kostroma region	196.01	57.69	138.32	8.51
10	Krasnodar region	194.62	43.06	151.57	22.50
11	Vladimir region	179.48	45.13	134.34	6.27
12	Novgorod region	178.94	40.61	138.33	5.64
13	Arkhangelsk region	176.59	40.02	136.58	6.56
14	Smolensk region	176.55	33.81	142.75	4.55
15	Ivanovo region	176.24	45.56	130.68	6.97

**The types and combinations of drugs discovered in this paper.**

- Distribution of the sum of all drugs represented in the market by the regions (grams per 100,000 population).
- Distribution of opiates represented in the market by the regions (grams per 100,000 population).
- Distribution of cannabinoids represented in the market by the regions (grams per 100,000 population).
- Distribution of drug stimulants represented in the market by the regions (grams per 100,000 population).
- The distribution of cocaine is represented in the market by the regions (grams per 100,000 population).

## Discussion

### Principal Findings

Considering the COVID-19 restrictions, the illicit nature of the studied substances, and their effects on people's health and well-being, we discovered all the proposed questions in this research. First, we learned how many drugs, in grams per 100,000 population, were found on the “shelves” of the Hydra dark market on a subnational level. In 2019, on an average day, the total quantity of drugs available for purchase on the darknet, in batches of 5 g or less, amounted to 124 kg. It was a period of COVID-19 restrictions, so we have data on the decreasing amounts of drugs distributed monthly in Russia during COVID-19 restrictions. A strong and significant correlation between the quantities of heroin for sale and lethal overdoses or fatal poisoning was established.

The higher amounts of cannabis products represented on the marketplace in the regions of Russia, the lower opiate overdose, and other harmful circumstances with significant negative correlations found. The supply of cannabis is about 10 times higher than opiates in terms of weight on the market.

A positive relation between higher population density in the regions and the level of HIV among injection drug users was found. The average amount of drugs per 100,000 people in Russia varies widely between regions. Law enforcement strategies, drug trafficking routes, local drug manufacturing, socioeconomic conditions, and drug usage culture may all play a role in this diversity. Unlike more rural or sparsely populated areas, drug use tends to be more prevalent in densely populated cities like Saint Petersburg and Moscow, like the Chechen Republic. Increased accessibility, larger populations, or different socioeconomic conditions could all be to blame.

Marijuana usage is widespread and accounts for a disproportionate share of all illicit drug consumption. It accounts for the typical drug amounts in most of the regions. This aligns with prevalent beliefs worldwide, as cannabis is the most widely used illicit substance.

Opiate usage is widespread in some areas, although it is significantly less common than cannabis use. Compared to other regions, Krasnodar's rate of 22.50 g of opiates per 100,000 people is exceptionally high.

Substances other than cannabis are widely used worldwide, as shown by the ”grams without cannabis“ column. Evidence of a varied drug market includes the prevalence of substances other than cannabis in quantities that outweigh cannabis alone. The distribution of the supply of opiates by regions of Russia is more concentrated in the European and southern parts of the country.

The Republic of Karelia and the Chukotka have higher drug use rates than other regions of similar populations. This could indicate that there are specific regional influences on drug use.

### Comparison to Prior Works

Much of the existing academic research [10,17-20] has focused on the content of listings on anonymous darknet markets and forums, and this body of work has already addressed related questions about the logistics of the drug trade: its supply chains and what illicit profits were made using Bitcoin blockchain transactions investigations, for example. Previous research has also been conducted on customer reviews from anonymous digital marketplaces, providing insights into consumer behavior, market dynamics, and vendors' perceived quality and reliability in these clandestine environments. In comparison, for the first time, our study uses direct dark web parsing to dig the drug merchandise and its supply amounts on the market.

### Strengths

The study uses the internet or darknet data for public health research, providing a unique insight into the scale and nature of drug sales. Python scraping from a major darknet marketplace offers a novel technique to understand drug availability and consumption patterns, which are typically difficult to assess using traditional public health tools.

The developed research method allows us to connect darknet drug sales data with official health, crime, and court information. This will help us better understand and predict the impact of hidden drug sales on public health. Our analysis links darknet drug data with published health indicators, such as overdose deaths, HIV cases related to drug use, and drug-related convictions, filling a gap in existing research.

Analysis has implemented geospatial analysis using darknet data aggregated by Russian regions and categorizing drug amounts, enabling specific public health policy making and interventions to help in the identification of regional variations in drug use and health impact.

Advanced data visualization techniques, such as interactive maps and correlation matrices, depict complex relationships between the darknet drug trade and health outcomes.

The open-source nature of all analytical instruments (Python, R, PHP [hypertext preprocessor, a scripting language]) allows further research to repeat or continue our study on other darknet platforms to investigate other regional specifics.

### Limitations of the Study

We have discovered, downloaded, and studied only 1 large darknet marketplace, “Hydra,” and selection and generalization biases can appear. The specific drug's popularity on Hydra might be inflated due to vendor marketing strategies rather than real user preference. This bias can be fixed by incorporating other prevalent drug distribution methods, such as traditional routes, novel platforms like social networks, and messaging services like Telegram and other darknet marketplaces, and by including the estimates from the other data sources.

The study's scope is limited by data scope, reliability, and reliance on the darknet marketplace. There is no way to fix this because Hydra was shut down in 2022, and new marketplaces were established. But we have all the data for the last 2 years when Hydra worked.

The generalizability is specific to Hydra and may not apply to other darknet markets or regions. Russia's unique socioeconomic context, for instance, influences drug trade dynamics differently than in other countries. Our proposed research approach can fix this by implementing it in other marketplaces in other countries.

### Future Directions

It is possible to implement our medical Internet research methodology with proposed open-source analytical and programming instruments for studying another darknet marketplace in other countries, such as Canada or the United States, and combining found indicators with official statistics, mortality, mortality registry, and observational studies data, and police and courts data to find matches with other relevant variables. This approach can cover the needs in public health research of the vulnerable populations and their hidden drug preferences with health circumstances and direct harm of illegal drugs on local, national, and global levels. We are encouraging future researchers to use our cascade of methods for sharpening and improving existing official and published data on every level.

Another future direction can be cluster analysis, using our research database, whereby people's incomes, group regions, and correlations are seen inside these clusters. Investigating clusters of regions aggregated by population-specific morbidity and mortality patterns also seems interesting and promising.

Our research database contains geospatial data, which allows us to analyze separate cities, towns, or even villages in the Russian Federation if such a question is raised or funding is found.

### Conclusions

Our analysis of drug supply and harmful effects across various regions of Russia has revealed stark differences in the levels and types of drug consumption. There is significant regional variability in drug usage, with the most urbanized areas like cities Saint Petersburg and Moscow experiencing higher levels of drug usage than the more rural or less populated regions. The higher income, digitalization of the citizens, and higher education levels can explain this.

Cannabis is the most widely used drug across all regions of Russia. Despite this, noncannabis drugs remain prevalent across all regions, hinting at a diverse drug market in the country. Opiates, while representing a smaller portion of overall drug use, have a significant presence, particularly in the Krasnodar region, which warrants further investigation.

Specific regions, such as the Chukotka Autonomous District and the Republic of Karelia, exhibit relatively prominent levels of drug usage compared to the other areas with similar demographic attributes, suggesting the influence of unique local factors on drug consumption patterns.

This study underscores the importance of a region-specific approach to drug policy in Russia. Policy makers should consider the unique contexts of each region, including the prevalent types of drugs, socioeconomic factors, and local attitudes toward drug use. While nationwide policies can provide a broad framework for combating drug use and related HIV and its associated harms, region-specific strategies are critical for effectively addressing local realities.

The patterns revealed in this analysis are not static; they can shift over time due to changes in drug supply, law enforcement efforts, and societal attitudes. Continued monitoring is essential to ensure drug policies remain responsive to the current situation.

The drug scene in Russia is a multifaceted issue that requires nuanced, data-driven, and region-specific strategies. A one-size-fits-all approach is unlikely to address each region's unique realities effectively. By recognizing and accounting for regional differences, policy makers can design and implement more effective strategies to combat drug use and mitigate its harms.

While this study provides valuable insights into the relationship between darknet drug sales and public health outcomes, it is important to note the limitations associated with using data from a single darknet marketplace (Hydra) and the potential biases in official statistics. These factors should be considered when interpreting the findings, and future research should aim to incorporate data from multiple sources and marketplaces to validate these results.

## Appendix

Multimedia Appendix 1 Additional table.

## Data Availability

All secondary data are available on the web [27-29]. The primary database can be requested by email from corresponding author of this paper by email: soshn1s@cmich.edu.
