# Supplementary material for: Association of Drugs for Sale on the Internet and Official Health Indicators: Darknet Parsing and Correlational Study
Source: JMIR Form Res. 2024 Nov 15;8:e56006. doi: 10.2196/56006 (PMC11607563; doi:10.2196/56006)
Supplement: Multimedia Appendix 1 [file formative_v8i1e56006_app1.docx]

**Table 2.** The average grams at the moment of parsing of drugs presented by the Russian region per 100,000 population of the area. Ranged decreasing by grams of all drugs.

|  | region | grams all | grams cannabis | without cannabis | grams opiates |
| --- | --- | --- | --- | --- | --- |
| 1 | Leningrad region | 401,68 | 88,27 | 313,41 | 19,51 |
| 2 | Moscow region | 342,60 | 88,70 | 253,90 | 8,74 |
| 3 | Kaluga region | 287,93 | 70,40 | 217,53 | 8,48 |
| 4 | Saint Petersburg | 276,83 | 71,87 | 204,96 | 10,26 |
| 5 | Republic of Karelia | 250,11 | 62,97 | 187,14 | 10,97 |
| 6 | Tver region | 222,24 | 52,22 | 170,02 | 7,60 |
| 7 | Chukotka | 221,68 | 52,08 | 172,42 | 18,49 |
| 8 | Kaliningrad region | 209,60 | 71,24 | 138,36 | 6,04 |
| 9 | Kostroma region | 196,01 | 57,69 | 138,32 | 8,51 |
| 10 | Krasnodar region | 194,62 | 43,06 | 151,57 | 22,50 |
| 11 | Vladimir region | 179,48 | 45,13 | 134,34 | 6,27 |
| 12 | Novgorod region | 178,94 | 40,61 | 138,33 | 5,64 |
| 13 | Arkhangelsk region | 176,59 | 40,02 | 136,58 | 6,56 |
| 14 | Smolensk region | 176,55 | 33,81 | 142,75 | 4,55 |
| 15 | Ivanovo region | 176,24 | 45,56 | 130,68 | 6,97 |
| 16 | Crimea | 168,37 | 43,88 | 124,49 | 5,51 |
| 17 | Kamchatka Krai | 167,54 | 30,16 | 137,38 | 3,92 |
| 18 | Pskov region | 163,35 | 38,95 | 124,40 | 6,28 |
| 19 | Sevastopol | 160,40 | 53,84 | 106,56 | 4,52 |
| 20 | Moscow | 159,52 | 42,59 | 116,93 | 4,42 |
| 21 | Tula region | 151,30 | 31,47 | 119,83 | 4,40 |
| 22 | Komi Republic | 143,41 | 35,54 | 107,87 | 5,20 |
| 23 | Kirov region | 139,67 | 38,57 | 101,10 | 5,36 |
| 24 | Bryansk region | 124,34 | 29,42 | 94,91 | 3,96 |
| 25 | Sverdlovsk region | 121,39 | 28,20 | 93,18 | 4,50 |
| 26 | Murmansk region | 116,99 | 23,37 | 93,62 | 6,96 |
| 27 | Nenets Autonomous | 115,16 | 27,21 | 90,89 | 11,22 |
| 28 | Ryazan Oblast | 112,46 | 27,46 | 85,00 | 4,51 |
| 29 | Vologda Region | 111,02 | 26,70 | 84,32 | 4,59 |
| 30 | Rostov region | 109,23 | 31,50 | 77,73 | 3,54 |
| 31 | Mari El Republic | 105,85 | 29,63 | 76,22 | 4,15 |
| 32 | Stavropol region | 103,42 | 25,71 | 77,71 | 4,77 |
| 33 | Samara Region | 99,21 | 26,81 | 72,41 | 2,84 |
| 34 | Novosibirsk region | 97,19 | 28,31 | 68,88 | 4,85 |
| 35 | Magadan Region | 94,81 | 21,51 | 73,31 | 8,62 |
| 36 | Voronezh region | 92,76 | 22,69 | 70,07 | 4,16 |
| 37 | Tyumen region | 89,49 | 27,06 | 62,43 | 4,42 |
| 38 | Nizhny Novgorod | 88,23 | 20,68 | 67,55 | 4,53 |
| 39 | Tomsk region | 87,74 | 19,37 | 68,37 | 2,98 |
| 40 | Perm region | 86,77 | 22,69 | 64,08 | 3,13 |
| 41 | Lipetsk region | 82,80 | 20,06 | 62,74 | 2,55 |
| 42 | Ulyanovsk region | 78,58 | 21,02 | 57,56 | 6,52 |
| 43 | Bashkortostan | 76,51 | 17,83 | 58,67 | 1,99 |
| 44 | Irkutsk region | 75,63 | 17,05 | 58,57 | 3,07 |
| 45 | Sakhalin Region | 75,47 | 14,80 | 60,67 | 8,34 |
| 46 | Belgorod region | 72,17 | 16,68 | 55,49 | 2,59 |
| 47 | Oryol Region | 70,91 | 17,91 | 53,00 | 3,40 |
| 48 | Chelyabinsk region | 70,86 | 20,30 | 50,56 | 2,24 |
| 49 | Republic of Tatarstan | 69,41 | 16,00 | 53,41 | 2,40 |
| 50 | Tambov Region | 68,57 | 18,70 | 49,87 | 1,95 |
| 51 | Udmurtia | 67,38 | 15,05 | 52,34 | 1,93 |
| 52 | Karachay-Cherkess | 67,04 | 18,94 | 48,61 | 5,47 |
| 53 | Kemerovo region | 61,72 | 14,75 | 46,97 | 3,23 |
| 54 | Chuvash Republic | 61,37 | 15,82 | 45,55 | 2,61 |
| 55 | Saratov region | 58,25 | 16,56 | 41,69 | 1,30 |
| 56 | Kursk region | 57,79 | 17,20 | 40,59 | 2,58 |
| 57 | Krasnoyarsk region | 57,53 | 15,18 | 42,35 | 1,99 |
| 58 | The Republic of Sakha | 53,09 | 12,97 | 40,12 | 2,77 |
| 59 | Primorsky Krai | 49,14 | 8,89 | 40,25 | 1,49 |
| 60 | Orenburg region | 48,33 | 12,35 | 35,98 | 1,22 |
| 61 | Altai region | 47,20 | 10,31 | 36,89 | 4,56 |
| 62 | Penza region | 47,01 | 11,55 | 35,46 | 1,92 |
| 63 | Kabardino Balkar | 45,78 | 9,86 | 35,92 | 2,19 |
| 64 | Amur region | 45,49 | 9,00 | 36,49 | 2,51 |
| 65 | Khakassia | 45,37 | 9,23 | 36,39 | 2,83 |
| 66 | North Ossetia - Alania | 40,52 | 10,56 | 30,24 | 2,43 |
| 67 | Volgograd region | 40,11 | 12,29 | 27,82 | 1,02 |
| 68 | Khabarovsk region | 38,77 | 7,48 | 31,29 | 0,84 |
| 69 | Kurgan region | 38,18 | 8,76 | 29,66 | 1,20 |
| 70 | Yaroslavl region | 37,25 | 8,52 | 28,73 | 2,13 |
| 71 | Jewish Autonomous | 36,61 | 8,06 | 28,99 | 3,08 |
| 72 | Republic of Kalmykia | 36,54 | 10,43 | 26,38 | 4,28 |
| 73 | Yamalo-Nenets | 32,01 | 7,72 | 24,50 | 2,51 |
| 74 | Altai Republic | 30,39 | 6,57 | 24,18 | 2,74 |
| 75 | Ingushetia | 29,74 | 6,56 | 23,35 | 4,16 |
| 76 | KhantyMansi | 28,92 | 7,09 | 21,83 | 3,46 |
| 77 | Republic of Adygea | 28,36 | 6,06 | 22,30 | 4,71 |
| 78 | Omsk region | 25,31 | 6,34 | 18,97 | 1,06 |
| 79 | Mordovia | 23,16 | 5,37 | 17,94 | 2,12 |
| 80 | Dagestan | 22,08 | 5,29 | 16,94 | 1,14 |
| 81 | Zabaykalsky Krai | 20,74 | 4,25 | 16,71 | 2,36 |
| 82 | Astrakhan region | 20,56 | 5,94 | 14,78 | 1,09 |
| 83 | Buryatia | 19,64 | 6,24 | 13,40 | 0,35 |
| 84 | Tyva Republic | 12,99 | 3,98 | 9,72 | 2,28 |
| 85 | Chechen Republic | 7,56 | 1,90 | 5,77 | 0,53 |
